# Contemporary Strategies and Current Trends in Designing Antiviral Drugs against Dengue Fever via Targeting Host-Based Approaches

**DOI:** 10.3390/microorganisms7090296

**Published:** 2019-08-28

**Authors:** Foysal Ahammad, Tengku Rogayah Tengku Abd Rashid, Maizan Mohamed, Suriyea Tanbin, Fazia Adyani Ahmad Fuad

**Affiliations:** 1 Department of Biotechnology Engineering, International Islamic University Malaysia, Kuala Lumpur 50728, Malaysia; 2 Virology Unit, Institute for Medical Research, Jalan Pahang, Kuala Lumpur 50588, Malaysia; 3 Faculty of Veterinary Medicine, Universiti Malaysia Kelantan, Locked Bag 36, Pengkalan Chepa, Kota Bharu 16100, Kelantan, Malaysia

**Keywords:** dengue virus (DENV), antiviral drugs, drug targets, DENV host factors, host metabolism, DENV inhibitors, arthropod-borne viruses

## Abstract

Dengue virus (DENV) is an arboviral human pathogen transmitted through mosquito bite that infects an estimated ~400 million humans (~5% of the global population) annually. To date, no specific therapeutics have been developed that can prevent or treat infections resulting from this pathogen. DENV utilizes numerous host molecules and factors for transcribing the single-stranded ~11 kb positive-sense RNA genome. For example, the glycosylation machinery of the host is required for viral particles to assemble in the endoplasmic reticulum. Since a variety of host factors seem to be utilized by the pathogens, targeting these factors may result in DENV inhibitors, and will play an important role in attenuating the rapid emergence of other flaviviruses. Many experimental studies have yielded findings indicating that host factors facilitate infection, indicating that the focus should be given to targeting the processes contributing to pathogenesis along with many other immune responses. Here, we provide an extensive literature review in order to elucidate the progress made in the development of host-based approaches for DENV viral infections, focusing on host cellular mechanisms and factors responsible for viral replication, aiming to aid the potential development of host-dependent antiviral therapeutics.

## 1. Introduction

Dengue is an important arthropod-borne viral infectious disease caused by any one of the four-dengue virus (DENV-1 to -4) viral serotypes. The antigenically distinct but closely related serotypes of DENV belong to the genus Flavivirus, family Flaviviridae. Its rapid and intense spread is noted in most of the world’s tropical and subtropical regions, which has led to its categorization as an emerging infectious disease [1,2]. The genus includes more than 70 small-enveloped viruses related to Japanese encephalitis (JEV), Zika viruses (ZIKV), West Nile (WNV), yellow fever virus (YFV), DENV, or tick-borne encephalitis (TBEV), and other medically-important arboviruses [3]. Most importantly, DENV is endemic in 112 countries, and incidence of infection has risen 30-fold over the last five decades [4]. More than one-third of the world population is at risk of infection, and it is estimated that ~400 million individuals suffer annually because of DENV infection [5]. DENV infection results in varying degrees of clinical signs and symptoms (asymptomatic or only mildly symptomatic). In the case of dengue fever (DF), patients may experience headache, myalgia, rash, leukopenia, arthralgia, retro-orbital pain, and hemorrhagic manifestations. Thereafter, patients suffering from dengue hemorrhagic fever (DHF) may develop petechiae, bruising, thrombocytopenia, and shock. Ultimate disease signs include rapid or weak pulse, manifestations of hypotension and clammy skin, and finally cold and restlessness in dengue shock syndrome (DSS) [6]. 

However, specific therapeutics are presently not available for DENV infections [7], necessitating an improved approach for re-mediating this global burden. In this review, we will explore the host factors or targets that influence DENV replication, focusing on the factors that can potentially be utilized in a new process that may help alleviate this global burden.

### 1.1. History

As one of the major public health concerns, the term “dengue” is believed to originate from the Swahili word “ki-dinga pepo”, which means “cramp-like pains, produced through the agency of an evil spirit” [8,9]. Joint fever, a type of dengue-like disease, was first recorded during the epidemics in Batavia (Jakarta) and Cairo in 1779. Subsequent epidemics (1780 to 1901) were reported in Philadelphia, Zanzibar, Calcutta, the West Indies, and Hong Kong. Where the relevant factors considered for all these epidemics were thought to be DENV, many others believed that it was not only dengue, but that the Chikungunya virus was also responsible for the epidemics [10,11]. The epidemic capability of dengue acquired a broad geographic distribution before the 18th century; however, the actual causes of dengue infection remained unknown until 1906 [12]. In 1907, Ashburn and Craig provided the first data indicating the filterable, ultramicroscopic character of an etiological agent, namely dengue, which is transmitted by a true vector *Aedes aegypti* [13]. Cleland et al. (1919) and Siler et al. (1926) subsequently confirmed that the DENV transmission process was akin to the “jungle cycle” of the yellow fever virus [14,15,16]. From 1922 to 1945, many outbreaks were recorded in the United States, Australia, Greece, and Japan. In 1922, it was estimated that 1 to 2 million individuals in the southern United States were affected. In the three decades after World War II, dengue epidemics occurred sporadically in Central America and the Caribbean basin [17]. Socio-economic conditions after the war resulted in poor immunity in most populations and contributed to an increased dengue incidence throughout the world, especially in Southeast Asia [18]. From 1943 to 1944, a major breakthrough occurred in the treatment of dengue fever, when Dr. R. Kimura (Japan) and Dr. S. Hotta (Hawaii) isolated and identified the DENV strains DENV-1 and DENV-2 [19]. In 1953, the DENV-3 and DENV-4 strains were first isolated from infected individuals in the Philippines and Thailand [20].

### 1.2. Vector and Non-Vector Transmission of Dengue

In most cases, DENV transmission is accomplished by the primary vectors, whereby *A. aegypti* and *Aedes albopictus* have long been recognized as hosts for the viruses [21,22]. *A. aegypti* is the most common primary epidemic-causing and predominant vector of dengue [6]. Other species that act as secondary vectors for carrying DENV include *Aedes scutellaris*, *Aedes africanus* (subgenus Stegomyia) and *Aedes niveus* (subgenus Finlaya), which are considered as sylvatic vectors. Meanwhile, other species such as *Aedes taylorior*, *Aedes furcifer* (subgenus Diceromyia), as well as *Aedes mediovittatus* (Gymnometopa) and *Aedes triseriatus* (Protomacleaya) also play key roles as secondary dengue vectors for carrying dengue infectious virus [22,23].

The non-mosquito DENV transmission route is infrequent and accidental, but it has great importance for physicians. Several authors have reported cases of transmission without a mosquito vector, which occurred in different ways, including through needles, congenitally, through mucocutaneous exposure, and through bone marrow transplants [24]. DENV infection of healthcare workers usually occurs as a result of needle injuries [25,26,27,28,29], while vertical transmission is responsible for dengue fever cases in infants born to a DHF-diagnosed mother [30]. A child from Puerto Rico attained DENV-4 via bone marrow transplant and subsequently died [31], and DENV-3 was transmitted to a healthcare worker through blood (mucocutaneous exposure) [32]. These are only a few examples of non-vector dengue transmission. 

### 1.3. Transmission Process 

**Figure 1:**
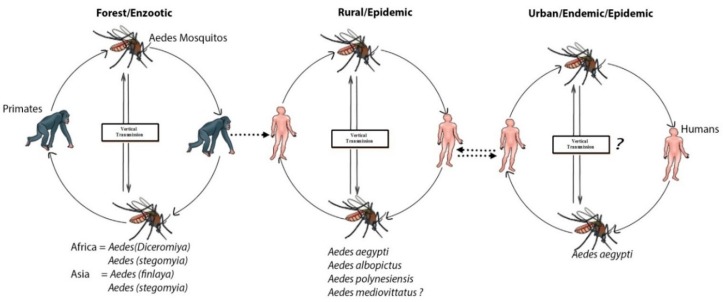
Sylvatic transmission of dengue virus (DENV) from lower primates to humans: Serotypes of DENV-1–4 emerged independently via the sylvatic cycle and subsequently disseminated among human populations. The forests of Southeast Asia and West Africa maintained the sylvatic cycle and supported contact with human populations, leading to an urban endemic/epidemic cycle between *Aedes* mosquitoes and human repository hosts.

A few hundred years ago, dengue was primarily a sylvatic disease. The sylvatic cycle is ecologically and evolutionarily-distinct from the human transmission cycle, causing sporadic outbreaks in humans [33,34]. A sylvatic cycle that serves as an enzootic cycle involving canopy-dwelling *Aedes* mosquitoes and lower primates for dengue transmission has been well-documented in the rainforests of western Africa and Southeast Asia, including Peninsular Malaysia and eastern Senegal [6,33]. *Aedes luteocephalus*, *A. furcifer*, and *A. taylori* were the principal transmission vectors in Africa [35,36,37,38,39], and the primatophilic canopy-dwelling mosquitoes of the *A. niveus s.l*. complex—a group that includes *Aedes pseudoniveus*, *Aedes subniveus*, *Aedes vanus*, *Aedes albolateralis*, *Aedes niveoidesan*, and *Aedes novoniveus*—were the principal vectors in Asia (Figure 1) [40]. In other locations in tropical Africa and Asia, probable rates of sylvatic DENV transmission to humans are unknown, but appeared to be minimal [41,42]. In North America, several species of new-world non-human primates were found to be resistant to DENV infection, and no cases of sylvatic DENV transmission were recorded. Similarly, no evidence of enzootic circulation through lower primates have been found in Panama [43]. However, seroconversions among indigenous Ayoreo Indians living in an isolated forested region of Bolivia, where *A. aegypti* is absent, recommend that sylvatic DENV transmission may happen in the region [44]. Cross-species transmission of DENV in rural areas of Africa and Asia from non-human primates to humans tends to occur when great numbers of the enzootic vector(s) are present [45]. The sylvan environments in rural areas of Africa and Asia can be a source of an abundant amount of primary vector *A. albopictus*, which is responsible for the transfer of the virus into human habitats [46]. Endemic/epidemic cycles involved the human host, and viruses are transmitted mainly by *A. aegypti*, *A. albopictus*, and other mosquitos that serve as secondary vectors, such as *A. mediovittatus*, *Aedes polynesiensis* and other members of *Aedes scutellarin* [47,48].

DENV-infected small *A. aegypti* bite humans and thus initiate DENV transmission. These mosquitos lay eggs and rest indoors in coconut shells, artificial containers, vases with wastewater, and old automobile tires found in and around homes. At dawn, 2–3 h after sunrise, as well as during sunset (in daylight hours), adult mosquitoes prefer to feed on humans, to which these periods are known as the “two peaks of biting activity”. *A. aegypti* females very often feed on several persons, thus rapidly accelerating the transmission of DENV [49]. After transmission into the human host and following a 3 to 14-day incubation period, most affected individuals enter a 2 to 10-day acute febrile period and experience nonspecific signs and symptoms. During this viremic stage, other biting mosquitoes become infected and viruses circulate in the peripheral blood.

## 2. Targeting Host as an Antiviral Approach

The significance of DENV and its mechanism of interaction with host factors must be fully understood for proper morphogenesis of DENV for targeting host processes as an antiviral therapy.

The virus depends on the host machinery to complete their life cycles. For example, RNA replication of the hepatitis C virus (HCV) depends on the human homologue of the 33-kDa “vesicle-associated membrane protein-associated protein (hVAP-33)”, Golgi-specific brefeldin factor 1 (GBF1)—a type of resistant guanine nucleotide exchange factor, and host geranylgeranylated proteins and fatty acids [50,51]. Meanwhile, the human immunodeficiency virus (HIV) utilizes host C–C chemokine receptor type 5 (CCR5) and C–X–C chemokine receptor type 4 (CXCR-4)—chemokine receptors as mediators of HIV infections [52,53,54]. The influenza virus requires post-entry steps for its replication and, for that purpose, it utilizes the host’s important nuclear components, proteases, and the calcium/calmodulin-dependent protein kinase IIb (CAM K2B) [55,56]. On the other hand, West Nile virus (WNV) replication is associated with intracellular membrane rearrangements, and these processes are related to host fatty acid metabolic pathways, as well as membrane re-modelling of the host [57,58]. DENV is not exceptional from the other viruses—it also depends on the host machinery to complete its life cycle. In order to understand DENV host dependent factors, a genome-wide screen to explain DENV host dependency for their life cycle in *Drosophila melanogaster* (host) cells has been published [59]. Later, different conservative genome-wide screens of DENV identified different host-dependent factors responsible for their replication, where metabolic pathways, receptors and attachment factors, host proteins or enzymes, host immune factors, and anti-inflammatory pathways are most commonly found to influence DENV replication and infection. 

Different host and viral factors play a crucial role in promoting more severe dengue cases. This may occur via two routes: (i) Severe diseases occurring along with secondary infections, where a heterologous antibody virus (IgG–DENV) complex forms to FcγR receptors on the macrophage and aid in amplifying the infections [14]. During this time, antibodies come from primary infections with different serotypes. (ii) Secondly, the amplified infections aid in the increased viral load leading to an immunopathogenic response. From the hypothesis, we can understand that DENV serotypes are propagated in endemic areas, and pre-existing immunity to one serotype does not defend against infection with other serotypes, as some serotypes are more virulent than other serotypes and may enhance the severity of disease [23]. Hence, evaluation of the host and viral factors (e.g., isoform of enzymes, serotypes of virus) must be considered carefully when choosing host pathway as a target for anti-DENV therapy that could play a role in the progression of severe dengue cases in the frame of all the four DENV serotypes. Prior to evaluation, sometimes targeting the factors is very difficult, and may be toxic for the host. Difficulties in attenuation, lack of stability, less broad, potent, and durable immune response are some of the biggest drawbacks for targeting host factors as potential antiviral therapeutics [36]. However, much potential exists in targeting the host factors for the invention of antiviral drugs, despite the factors discussed above.

## 3. Targeting Host Metabolic Pathway

Viral infections can modify many physiological as well as metabolic pathways. Metabolic changes include lipid metabolism, in addition to stimulation of glycolytic pathways toward an energetically favorable state, which modifies membrane lipid and other composition for viral replication and virion envelopment. Targeting intracellular metabolic pathways and their pharmacological inhibition can reduce DENV RNA synthesis and infectious virion production [60], which may serve as successful DENV antiviral strategies. For example, lipid and glucose metabolic pathways are necessary for every step in the replication cycle of DENV. Both steps in the replication cycles of DENV can be inhibited by different pharmacological agents, and the agents/inhibitors developed mainly target the host factors that mediate lipid synthesis, lipid and glucose metabolism, and trafficking pathways. Despite this, targeting host glucose and lipid metabolism and trafficking as an antiviral strategy by blockade of entire pathways may be limited because of host toxicity [50]. Knowledge of the molecular details of lipid and glucose metabolic pathways, regulatory enzymes of the pathways and metabolic function in replication, and the mechanisms by which specific glucose and lipids are generated during DENV infection, as well as its trafficking to the relevant factors, will help to enable more targeted antiviral strategies without creating any toxic effects on the host cell.

### 3.1. Targeting the Host Glycolytic Pathway

**Figure 2:**
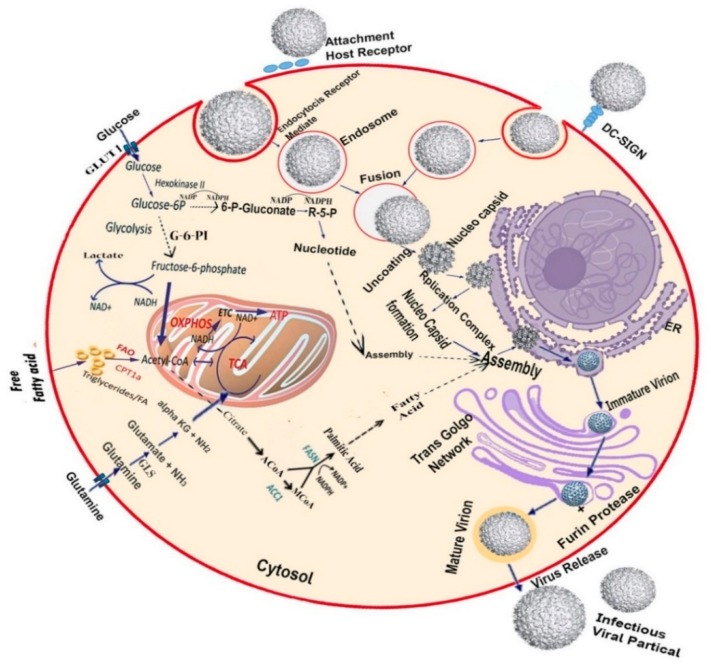
Host metabolic pathways are necessary for DENV replication: As an enveloped virus, DENV requires fatty acids for replication and virion envelopment, resulting in virus-mediated modifications in the host cellular systems. These modifications lead to alterations in glucose and glutamine metabolism, as well as fatty acid synthesis. At the primary level of infection, lipid droplets and nucleotides are reabsorbed into the endoplasmic reticulum (ER) and subsequently assemble with the DENV virus. Glucose uptake in DENV-infected cells may increase through the induction of the glucose transporter 4 (GLUT-4) or overexpression of glucose transporter 1 (GLUT-1) and hexokinase II (HK-II), the first enzyme of glycolysis. DENV infection alters glucose metabolism allosterically by up-regulation of glycolytic enzymes. Infected cells stimulate glycolysis to produce ATP through the tricarboxylic acid (TCA) cycle. It also generates citrate, which is a precursor of fatty acid biosynthesis. Glucose carbons are diverted and subsequently migrate to the cytoplasm from the TCA cycle through citrate. Exogenous glutamine uptake is increased in DENV-infected cells [5]. The TCA cycle is maintained by glutaminolysis enzymes that are induced by DENV, whereas imported glutamine was converted into α-ketoglutarate. Fatty acid and sterol synthesis are upregulated, so that acetyl-coenzyme A (AcCoA) can be used for fatty acid synthesis. Lipid synthetic enzymes are modified to generate a large amount of distinct membrane lipid [60,61]. Experimentally limiting glucose and fatty acid synthesis during DENV infection, along with limiting glutamine levels, can help prevent infections.

The host’s cellular metabolism provides the necessary energy (ATP), biosynthetic building blocks, and other important molecules required for viral replication. In DENV infection, a major change occurs in the central carbon metabolism, especially in glycolysis, whereby the expression of both glucose transporter I (GLUTI) and hexokinase II (HK-II) is up-regulated and glucose consumption is increased in DENV-infected cells. DENV activates the glycolytic pathway for viral metabolic requirements and life cycles, including energy, replication, and biosynthetic building blocks [60]. Glucose and glutamine serve as the main carbon sources in healthy cells and the tricarboxylic acid (TCA) cycle generates ATP using the oxidation of glucose via glycolysis. However, in some cases, glutamine serves as an ATP generator in the TCA cycle instead of glucose, so that it can be utilized for biosynthetic processes (Figure 2), such as in the case cancer cells and human cytomegalovirus (HCMV) cells [3,4,5,6]. As DENV activates the host glycolytic pathway for generating their necessary building blocks, pharmacologic regulation of glycolysis significantly blocks infectious DENV production. Krystal and colleagues reported that glycolysis inhibition through sodium oxamate and 2-deoxy-d-glucose (2DG) treatment can result in a significant reduction in DENV replication [5].

### 3.2. Targeting the Host Lipid Biosynthesis Pathway

As an enveloped virus, DENV stimulates the lipid biosynthesis pathway for essential membrane formation. Fatty acids, triglycerides, and other lipid compositions of the host are utilized by Flaviviruses for envelope formation. Membrane composition is not only required for the formation of the envelope but is also needed for inducing viral infection in many ways. Replication includes virion egress and assembly requires a great number of fatty acids and their derivatives that generate membrane. In DENV-infected cells, fatty acid synthesis is regulated to utilize acetyl-coenzyme A (AcCoA) for generating most distinct membrane lipids (Figure 2). DENV stimulates fatty acid biosynthesis by the help of the important cofactor fatty acid synthase (FASN), which was first identified through the DENV-2 replicon mediated siRNA screening [60,62]. Lipophagy is known as a type of selective autophagy that transports lipids for β-oxidation. Several studies indicate that DENV infection induces pro-viral autophagy [62,63,64,65,66]. The lipids accumulated in auto-phagosomes next transport to mitochondria, increasing the β-oxidation rate, which generates energy and plays a key role in lipophagy, thus assisting DENV replication. Additionally, NADPH that arises through β-oxidation utilizes a cofactor of FASN and it may stimulate the fatty acid synthesis for DENV replication. In DENV-infected cells, both fatty acid synthesis and lipophagy process take place at the same time. In contrast, both processes do not occur in natural cells at the same time [67]. Extant research has shown that pharmacological inhibition of FASN [68] and mevalonate diphosphate decarboxylase, an enzyme required for cholesterol biosynthesis [60], can decrease DENV production in host cells.

### 3.3. Targeting the Host Nucleoside Biosynthesis Pathway

All viruses require host nucleosides for replication. Host proteins associated with nucleoside biosynthesis can thus be targeted as anti-dengue therapeutics. Nucleotide guanosine 5′-triphosphate (GTP) pool depletion has emerged as a significant system for repressing Flaviviruses. Guanine biosynthesis can be inhibited through antiviral ribavirin [69]. Dihydroorotate dehydrogenase (DHODH) is a mitochondrial protein that catalyzes the oxidation of dihydroorotate to orotate. It is an essential enzyme in the de novo pyrimidine biosynthesis pathway. Available evidence indicates that using brequinar (a known DHODH inhibitor), an anti-metabolite in cancer and immune-suppression, can inhibit DENV type 1,2,3 (DENV-1,2,3) serotypes. However, it cannot inhibit DENV type 2 (DENV-2) variants because of resistance against brequinar, which was also cross-resistant to compound NITD-982 [70,71]. The NITD-982 analogue directly bound to the DHODH protein. A study also suggests that compound NITD-982 is also capable of inhibiting host DHODH [69]. An in vitro study of the compound shows a great potency against DENV serotypes, but the compound did not show any efficacy because of the exogenous uptake of pyrimidine from the diet in the DENV-AG129 mouse model (deficient in interferon alpha/beta and gamma receptor signalling). Targeting the enzymes that play a key role in supplying DENV nucleoside can therefore be effective for antiviral therapeutics.

## 4. Targeting Host Cellular Receptors and Attachment Factors

Several host factors at the cellular level play a key role in the DENV virus entry process, but attachment factors and receptors are deemed the most important factors. Molecules in mammalian cells can act as attachment factors and receptors. Dendritic cell-specific intercellular adhesion molecule-3-grabbing non-integrin (DC-SIGN) [72,73] and glycosaminoglycans (GAGs) [74] are the first line of attachment factors and receptors, while second-line molecular factors include the GRP-78—also known as binding immunoglobulin protein (BiP), the laminin receptor [75] and the T-cell immunoglobulin and mucin domain (TIM), and Tyro3, Axl, and Mer (TAM) receptors [76]. Glycosphingolipids (GSLs), chaperone-proteins, and undefined proteins have been reported as potential treatment candidates. Targeting host factors involved in DENV attachment can thus have a beneficial antiviral potential.

### 4.1. Heparin and Heparan Sulfate (Glycosaminoglycans)

**Figure 3:**
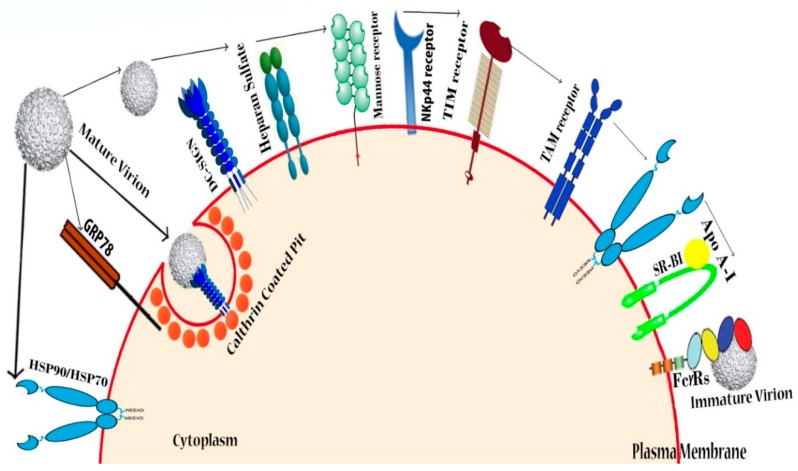
DENV receptors in human cells: DENV recognizes various types of receptors present in the host cells. Heparan sulfate was identified as the first molecule responsible for DENV entry into mammalian cells, while sulfated glycosaminoglycans—ubiquitous molecules on the cell surface—were found to play a role in mediating DENV attachment. CLEC4L (C-type lectin domain family 4, member L), CLEC4M (C-type lectin domain family 4, member M), and mannose receptor/CLEC13D/CD206 (cluster of differentiation 206) are some conventional C-type lectin receptors (CLRs) that have a high affinity towards high-mannose ligands involved in DENV entry into target cells, where C-type lectin domain family 5 member A (CLEC5A) lectin acts as a signaling receptor for releasing proinflammatory cytokine. Cell surface chaperones HSP-90, HSP-70 and GRP-78, all of which are part of the receptor complex, assist in the DENV binding. Lipid receptors phosphatydil serine (PtdSer), T-cell immunoglobulin and mucin domain (TIM), and Tyro3, Axl, and Mer (TAM) types of putative receptors are relevant for viral entry into the human 293 T-cell line [87]. Another receptor, SR-BI, is also responsible for lipoprotein-associated interaction with DENV and allows the virus to enter the host [76].

Heparan sulfate (HS) belongs to the family of glycosaminoglycans (GAGs). It initiates interactions between the DENV envelope protein and the host cell (Figure 3). HS also exhibits anti-DENV like molecular properties. For example, HS that was extracted from shrimp heads exhibits a strong inhibitory effect on infections caused by DENV [74]. Chondroitin sulfate, a curdlan sulfate that inhibits all DENV serotypes, has been found in baby hamster kidney 21 (BHK-21), Vero cells, and the rhesus monkey kidney cell (LLC-MK2) line [77,78]. Suramin [79,80], heparin [81], fucoidans [82], K5 polysaccharide from *Escherichia coli* [83], the heparan sulfate (HS) mimetic PI88 [80], α-d-glucan [84], GAG [85], and dextran sulfate (DS, MW > 500,000 Da) [85] are also able to inhibit DENV-2, whereas carrageenan [86] and DL-galactan hybrids extracted from red seaweed that lack cytotoxic effects and anticoagulant properties [86] can inhibit both DENV-2 and DENV-3 virus-infected cells (Table 1). Hence, targeting host heparin and heparan sulfate mimetics may act as potential antivirals that can aid in remediating the disease.

### 4.2. DC-SIGN

DC (dendritic cell) is a cell surface attachment factor present in every tissue [88]. It is known as Cluster of Differentiation 209 (CD209) and was found to regulate DC trafficking and T-cell synapse formation expressed by human immature dendritic cells in the plasma membrane capable of recognizing DENV. It not only recognizes DENV, but also allows its surface transport through the process of endocytosis, resulting in cell infectivity (Figure 3) [73,89]. Although the role of DC-SIGN in DENV entry remains controversial [90,91,92], many authors posit that DENV is handed over to another unidentified co-receptor for movement throughout the cells [90]. Findings yielded by a large number of studies indicate that DENV can recognize cell surface DC-SIGN, as well as move into the plasma membrane through various co-receptors. For example, HIV co-receptor CCR5 allows viral attachment, resulting in entry into the cell [89,93]. As DENV can pass to clathrin-coated structures (CCS) through virus receptor complexes [22], receptors that mediate DC-SIGN are potential targets for DENV antiviral treatment. Several compounds, such as glycomimetic DC-SIGN ligand and plant lectins from *Hippeastrum hybrid*, *Galanthus nivalis*, and *Urtica dioica* (Table 1), were found to act as strong inhibitors of DENV infection in DC-transfected cells. These findings can potentially be utilized in novel strategies aimed at enhancing the efficiency of a wide spectrum of antiviral therapies to block DENV virus uptake.

### 4.3. Other Possible Receptors

Viral attachment to the cell requires many sequential interactions with various receptors. DC-SIGN and glycosaminoglycans (GAGs) are known as the first line of attachment receptors. The second line of higher affinity receptors may then be recruited to permit DENV entry because of the diverse tissue tropism of the virus [94]. DENV uses two pathways to enter DCs, whereby infection can enter immature DCs through DC-SIGN, or through Fc gamma receptors (FcɣRs) in mature DCs. DCs expressed FcɣRs as the second line of a higher affinity receptor in host cells, and activation of FcɣRs in hematopoietic cells serves to remove antibody-opsonized antigens—including DENV—from the body circulation system. However, cross-reactive or sub-neutralizing levels of antibodies grant an alternative entry pathway of DENV, where DENV enters monocytes, macrophages, and dendritic cells through the activating FcɣRs [95]. DENV can enter cells via cellular attachment molecules’ TIM and TAM receptors [76], as both are able to recognize the apoptotic marker phosphatidylserine (PtdSer) and are responsible for the engulfment and removal of apoptotic cells. Since DENV is a virus that exposes PtdSer in its membrane, it naturally enters the cell as a PtdSer through direct binding of the TIM receptor or indirectly via the TAM receptor. Moreover, apoptotic marker PtdSer binds with TIM and TAM by the help of the growth arrest-specific 6 (Gas6) binder molecule (Figure 3) [96,97]. Cell surface chaperones, heat shock protein (HSP-90, HSP-70), and GRP-78 are known as a receptor complex, which allows DENV entry into human cells from hepatic, neural, and monocytic cells [75,98]. The interaction between apolipoprotein A-I and the scavenger receptor class B type I (SCARB1), also known as SR-BI, promotes DENV infections, necessitating further research in order to elucidate the functional importance of lipoproteins in dengue pathogenesis [87].

## 5. Targeting Host Proteins or Enzymes

Host proteins and enzymes play an integral role in Flaviviruses and are necessary for their entry into the host, as well as for replication and assembly. DENV dominates some processes to manipulate the host cell proteins and metabolic pathways. Post-translational modifications, especially the carbohydrate modification pathways (e.g., glycosylation), have been demonstrated as targets against Flaviviruses [94]. Toxicity and side-effects that were generated through the inhibition of proteins must therefore be carefully considered when targeting host proteins as antiviral therapeutics [99]. However, the potential of such compounds for screening purposes is tremendous. The host proteins are potential antiviral targets and have been shown in extant studies focusing on inhibiting such compounds so as to not be toxic for the host [100] (Table 1).

### 5.1. Targeting Host Protease 

Host protease is an effective target for antiviral drug development, as it is essential for virus replication. Host proteases, such as furin and signalase, have been used to cleave the DENV RNA genome co- and post-translationally and were translated as a polyprotein [101]. Correct processing can be used to generate the polyproteins essential for the viral life cycle [100,102]. Since host protease required for virus polyprotein formation is a basis of DENV replication, it can be a strong target for antiviral production. Protease furin is enriched in the Golgi apparatus of the host cell, where it assists in cleaving the DENV prM proteins, resulting in the formation of mature active forms of the virion M protein that plays an important role during DENV infections [99,103]. The signal peptidase on endoplasmic reticulum (ER) membrane cleaves the C/E-prM junctions [104]. It processes many secretory proteins, but the inhibition is likely to have side-effects. Nonetheless, recent research indicates that peptidomimetic furin luteolin inhibits the viral maturation process in an uncompetitive manner [105]. Peptide compound 45 and 46, as well as cavinafangin, have also been used as protease inhibitors in DENV-infected cells (Table 1). This finding indicates that further study is needed for the development of host proteases as an antiviral target.

### 5.2. Targeting Host Kinases 

Host kinases are involved in DENV assembly and secretion. Protein kinase inhibits the dengue replication cycle and, in the absence of a cytotoxicity cause, multilog decreases in the viral titer. Dasatinib and saracatinib (AZD0530) are inhibitors of the protein kinase c-Src [106]. Compound 16i is a kinase inhibitor that is ten times more potent than ribavirin. It can thus capture both the virus NS5-NS3 interaction and the host kinases c-Src/Fyn [107]. SFV785 and derivative compounds affect the neurotrophic receptor tyrosine kinase 1 (NTRK1) and MAP kinase-activated protein kinase 5 (MAPKAPK5) kinase activity and inhibit DENV propagation [108]. GNF-2 and imatinib inhibit DENV but are mediated by cellular Abl kinases [109]. Many compounds that inhibit kinase activity by regulating mitogen-activated protein kinase (MAPK or MAP kinase) have been developed. Examples of such compounds are CGP57380 that inhibits extracellular receptor kinase (ERK) and p38 pathways in DENV-2 infected cells; and PD98059, U0126, and FR180204 that inhibit the MAPK/ERK kinase (MEK); while AR-12 inhibits PI3K/JAKT pathway by expressing GRP-78 for all four DENV serotypes. Sunitinib and erlotinib, as well as isothiazolo[5,4-b] pyridines and Imidazo[1,2-b] pyridazine, are inhibitors of AAK1 and GAK pathways that inhibit DENV replication, whereas U0126 inhibits the ERK pathway to reduce the replication DENV-2 and -3 infected cells (Table 1). These findings provide pharmacological evidence that kinase has the potential to become a new class of antiviral target.

### 5.3. Glucosidase Inhibitors 

Glucosidase is a type of host enzyme, liable for viral maturation and proper folding. It initiates the process of glycosylation in N-linked oligosaccharides of the viral prM and E glycoproteins [110]. DENV structural protein prM and E glycoproteins are translocated into the host endoplasmic reticulum lumen (ER). During this time, a high mannose-rich oligosaccharide -Glc-3-Man-9-GlcNAc-2- (a total 14 residue core unit) is added co-translationally [111,112]. The resulting N-linked glycans are generated through the help of enzyme glucosidases I and II, where glucosidase I removes the terminal α-(1, 2) linked glucose from Glc-3-Man-9-GlcNAc-2, and glucosidase II removes the second and possibly the third terminal α-(1, 3) linked glucose residues from the Glc-3-Man-9-GlcNAc-2 oligosaccharide precursor, whereby the process is denoted “glucose trimming”. After that, it leaves the protein monoglucosylated and binds to the endoplasmic reticulum chaperones (calnexin or calreticulin) for proper folding [113]. As DENV prME heterodimer formation is not influenced by the inhibition of glucose trimming, it helps in generating a less stable complex characterized by reduced folding efficiency. It has been shown that the folding, stability, secretion, and activity of DENV glycoproteins in the ER depends on the trimming of these N-linked carbohydrates at N-130 and N-207 [110,112,114], thus rendering the responsible cellular glucosidase a potential host target. Castanospermine, a type of naturally occurring iminosugar, and N-nonyl-deoxy-nojirimycin (NNDNJ) isolated from *Bacillus*, are effective glucosidase inhibitors that have been indicated by both in vitro and in vivo studies in mice. The α-glucosidase inhibitor celgosivir has been shown to inhibit DENV, and treatment with celgosivir have shown to causing an improved survival rate in DENV infected mouse. The efficacy analyses were performed in patients with dengue fever [115]. The compound celgosivir is generally safe and well-tolerated but does not seem to reduce the viral load or fever burden in patients with dengue. Celgosivir derivative of 6-*O*-butanoyl is an oral pro-drug of castanospermine that can cause strong inhibition of DENV-1–4 [115,116]. Iminosugar drug UV-4, derived from deoxynojirimycin, was reported to decrease mortality in an “antibody-dependent enhancement” model of secondary DENV infection [117]. It is also noteworthy that α-glucosidase substrate mimics, such as CM 9 to 78 and CM 1018 (Table 1), are currently under development [118].

## 6. Targeting Host Immunity and Inflammatory Pathways

After the virus infects the host, cell signals are generated to block the dissemination of DENV. DENV causes acute disease without persistent infection. All virus strains have developed strategies that bypass the innate and adaptive immune response. Therefore, DENV does not escape the host defense mechanism. Innate immunity is known as the first line of antiviral defense mechanism that uses cytokine interferon I (IFN-I) for host defense purposes. It also results in the rapid activation of adaptive immune responses, resulting in the complete elimination of the virus [119]. To achieve this beneficial response, the immune system induces various factors and a series of gene expressions, including interferon-stimulated genes (ISGs), inflammatory responses, plasma, and vascular endothelium leakage, along with the disease progress factors both in infected and uninfected cells [120]. Hence, a better understanding of the host immune response during DENV infection and the evasion mechanisms would have great importance for potential antiviral production.

### 6.1. Targeting Host Immune Factors Involved in DENV Sensing

Viral pathogenic factors can be recognized through the pattern recognition receptors (PRRs) in the host cells. The endosomal toll-like receptors (TLRs) that recognize double-strand RNA (dsRNA) in endosomes, and the cytoplasmic receptor family complex form DEAD box, DEAH and the SKI proteins (DExD/H box), RNA helicases, Retinoic-acid inducible gene I (RIG-I) and Melanoma differentiation-associated protein 5 (MDA5)) that recognize intracellular double-strand dsRNA or single-strand viral RNA (ssRNA) [121], are the most important sensors in human cells that are implicated in detecting viral nucleic acids. After viral recognition by these two molecules, the interferon regulatory factors and the NF-kB (Nuclear factor kappa light chain enhancer of activated B cells) transcriptional molecules are activated, and these signaling cascades aid in generating IFN-α/β and inflammatory cytokines to activate the DC for an antiviral response. In the case of DENV, infected cells do not express themselves as a viral ligand sensed by retinoic-acid inducible gene I (RIG-I) and melanoma differentiation-associated protein 5 (MDA5), which is the reason why viral antibody is not detected by RNA sensors and C-type lectin domain family 5 member A (CLEC5A) does not block DENV infection [122]. Other important molecules such as toll-like receptors (TLR3) also sense dsRNA and can restrict DENV replication in different cell lineages [123,124,125]. DENV produces excess IFN-I (essential for DENV-induced production) through toll-like receptors 7 (TLR7) in plasmacytoid-DC, which can also restrict DENV replication in different cell lineages, as TLR3 [126]. Additionally, increasing inflammatory and humoral responses that decrease DENV replication have been found, such as when TLR3 and TLR7/8 agonists administrated into rhesus macaques aided in enhanced antiviral mechanisms during primary DENV infection [127]. Gene expression analysis indicates that RIG-I and MDA5 receptors promote the sensing ability in DENV-infected cells. The infected cells interact with these two receptors and stimulate interferon regulatory factor 3 (IRF-3) and the nuclear factor NF-kB that produces interferon beta (IFN-β) promoters, resulting in impaired replication of DENV. Additionally, DENV with double or single RIG-I/MDA5-deficient fibroblasts triggers both responses [128]. Furthermore, early detection of antibodies via RNA sensors (for examples RIG-I) enhanced DENV infection by tissue-resident mast cells that produce type IFN-I, along with chemokine ligands (CCL4, 5) and C-X-C motif chemokine ligands 10 (CXCL10) [129]. Consequently, human brain microvascular endothelial cells are infected with DENV, and rapid production of type-I IFN and proinflammatory cytokines were revoked after inhibition of RIG-I. Later, it was found that IFN-β production is induced by RIG-I, MDA5, and TLR3 sensors (major PRRs recognize innate responses to DENV infection), contributing to impairing DENV replication in vitro [130]. Activation of these major pattern recognition receptors (PRRs) by DENV generates a strong type of IFN-I response during human natural infections. Elevated levels of IFN-α for long periods in pediatric patients after the decrement period has also been reported [131]. Extant evidence (experimented upon mice) further indicates that DENV primary infection utilizes the interferon α/β receptor (IFNAR)-dependent (including STAT1-dependent and STAT1-independent) control mechanisms—where the STAT1 (Signal transducer and activator of transcription 1)-dependent mechanism controls the primary steps of infection, while the STAT1-independent mechanism controls the latter antiviral process—aiding virus propagation and disease control. In DENV primary infection, cells typically utilize both mechanisms [132,133]. Anti-CLEC5A blocks DENV by releasing pro-inflammatory cytokines and does not affect IFN-I production in the infected cells [134]. As CLEC5A blocks DENV infections, future studies are necessary to enhance CLEC5A activation.

### 6.2. Targeting Anti-Inflammatory Cytokine Populations

It has been demonstrated that excessive inflammation contributes to the pathogenesis of the severe form of dengue disease. Tumor necrosis factor (TNF)-α; interleukin (IL)-6, 8, 10; chemokine ligand (CCL)-2, 3; CXCL-8, 10; and interferon (IFN)-γ, all of which are responsible for primary and secondary DENV infection in humans, are excessively elevated and have been reported in patients with severe dengue disease [132,135,136,137,138,139,140,141]. These factors are activated by IFN-I and PRRs and were discussed in the preceding section. 

Cytokines are not responsible in causing any primary and secondary DENV infections in humans. Cytokine IFN-γ production in the host cell plays protective roles during primary DENV infection. In primary DENV infections, production of IL-12 and IL-18 proinflammatory cytokines precede IFN-γ release, and optimal IFN-γ production relies on the combined action of these two cytokines. For example, higher levels of IL-12 and 18 cytokines that are required for optimal IFN-γ production are usually recorded for DF patients, but in the case of DHF patients (Grade III and IV), the levels of this cytokine were non-detectable [142,143,144]. It has been demonstrated that IFN-γ controls nitric oxide synthase II-mediated nitric oxide production that assists the host in resistance against primary DENV infection [145], which was previously found to inhibit DENV replication [146]. Sustained IFN-γ production is necessary during the acute phase of illness to protect the host against fever and viremia [147]. With increased production of IFN-γ, the survival rates enhanced in DHF patients [138]. Hence, IFN-γ can be a potential target for the host to control DENV replication and resistance to infection. Enhanced proinflammatory cytokine TNF-α production is also associated with severity of dengue manifestation in humans. For example, T-cells isolated from patients are found to contain higher amounts of TNF-α after ex vivo stimulation with DENV antigens [148]. Hence, the blocking of enhanced proinflammatory cytokine TNF-α might reduce the pathology due to the primary [149], [150] and secondary [151] infections. The migration inhibitory factor (MIF) is indicative of a more severe disease form during primary DENV infections [138]. Experiments have shown that DENV primary infections were less severe in MIF^−^/^−^ mice, and they exhibited a significant delay in lethality, indicating that reduced proinflammatory cytokine levels (such as TNF-α) are correlated with lower viral loads at the initial phases of infection. Therefore, elevated production of the proinflammatory cytokines TNF-α and MIF during the host response to DENV infection favors more severe disease [152].

The chemokine system that plays a protective and pathologic role during DENV infections produces CXCL10 and activates CXCR3 (C-X-C chemokine receptor type 3) to improve host resistance against DENV infection [7]. Clinical studies in endemic areas indicated the presence of a correlation between DENV outcome and the level of CCL2, 3, 4 concentrations that were related to hypotension, thrombocytopenia, and hemorrhagic shock. Another study also found a link between CCL5 and DENV-induced hepatic dysfunction [135,139,153]. Reduced lethality rates, liver damage, alleviated leukocyte activation, and lower production of IL-6 have been found in chemokine receptor type 2 and type 4 (CCR2^−^/^−^and CCR4^−^/^−^) knockout mice with primary DENV infection. However, no difference in viral load has been found in the case of CCR-deficient mice. Hence, we can conclude that CXCR3 expresses protective host responses, but CCR2 and CCR4 cause infection rather than providing protection against DENV infection [154].

Cellular populations are also important for DENV infection. For example, cluster of differentiation 8 (CD8+) T cells are crucial for the control of viral replication, whereas an invariant natural killer T (iNKT) cell is important in the pathogenesis of dengue disease [155].

### 6.3. Targeting Host Plasma and Vascular Endothelium Leakage

Plasma leakage is an important factor in dengue disease progression and can cause DHF/DSS. Endothelium (primary fluid barrier) is changed by DENV, inducing edema and hemorrhage because of cell barrier permeability [200]. Vascular leakage can be blocked by FX06 (28-AA cleavage product), which decreases primary dengue infection. The protective effect of FX06 has been found to be elevated when combined with src/Fyn kinase. After activation, the 28-AA (28-Amino Acid) cleavage product Fyn dissociates from vascular endothelial and is combined with p190-Rho-GAP, an antagonist of RhoA activation. Thus, blocking vascular leakage by stabilization of endothelial cell development is important for DENV infection prevention [201]. The MIF inhibitor ISO-1 reduces permeability in the human hepatoma cell line. ISO-1, or the phosphoinositide 3-kinase (PI3K/AKT)/Ras-Raf-MEK-ERK/JNK signaling pathway, can be partially inhibited through the tight junction protein zonula occludens-1 (ZO-1) [202]. Chemokines CCL2 [168], leukocyte metalloproteinases 9 and 2 [203], and Box1 (HMGB1) [204] proteins are also involved in increasing vascular permeability. It has also been shown that type I-IFNs, IFN-β, VEGFR2 (Vascular Endothelial Growth Factor Receptor 2), and INF-α inhibit plasma leakage, where this process occurs with the help of endothelial stabilization [205].

### 6.4. Targeting Immune Factor Progress Disease after DENV Infection

Platelet-activating factor receptors (PAFR) released from macrophages, which were obtained previously from patients that were primarily infected with DENV-1, were found to be involved in the pathogenesis of severe dengue. The inflammatory response has also been demonstrated in DENV-2 virus infection, whereby PAF/PAFR was reported to interact with leukocytes and other cells [206].

Asunaprevir [192] and salidroside [191], extracts from *Uncaria tomentosa*, *Norantea brasiliensis* Choisy, *Uncaria guianensis* [196,197], promyelocytic leukemia protein intrinsic, Ivermectin [198], extracts from *Cissampelos pareira Linn* [198], and BST2/tetherin [199] are some of natural products that have advantages over synthetic drug design [207] and also tested as DENV inhibitors. Interferon type I [129], human heme oxygenase I, purinergic receptor P2X7 [195], and helicase with zinc linger 2 [194] are other examples of DENV inhibitors that have been experimentally studied to ascertain their dependence on host immunity, inflammation, pathogenesis of disease, and other disease progress factors (Table 1).

## 7. Conclusions and Remarks

Several remarkable points arise in the way of host inhibition processes that interrupt DENV replication along with infections. Host metabolic pathways serve as a source of energy, and molecular building blocks are required for the multiplication of DENV. For example, the primary glycolysis is conservatively required by DENV and exogenous glucose, and glutamine deprivation decreases DENV production, which could lead to the development of novel broad-spectrum antiviral therapies. The fatty acid metabolic pathway induces the activation of autophagy in DENV-infected cells by increased β-oxidation of fatty acids, and helps to bind with C proteins during virion assembly. An extensive body of research has been dedicated to the role of lipid and fatty acid metabolism during DENV infection, and the extant findings indicate that modulating lipid metabolisms in the host can be a viable anti-dengue therapeutic approach. Cellular nucleoside biosynthesis pathways of the host supply necessary nucleosides required for DENV replication. Hence, targeting the host nucleoside biosynthesis pathways can assist in blocking the essential functions of DENV as another avenue for antiviral drug development. 

In the case of viral infection, initial attachment to the target cell is necessary to continue the viral life cycle. This process can occur in DENV through the interaction between viral surface proteins and host attachment factors, or receptor molecules present at the host cell surface. These factors are responsible for the binding of a viral protein that leads to viral cell entry and subsequent genome release into the cytoplasm. If the early steps of DENV infection cycle can be blocked by targeting host attachment factors or receptor molecules, this would be significant progress in the development of antiviral drugs.

Without utilizing host proteins and enzymes, DENV would be unable to propagate rapidly in host cells. Numerous host proteins are found to be essential in DENV replication. For example, host proteases aid in the RNA genome cleavage and polyprotein formation, while host kinases help in DENV assembly, and glucosidase is used in DENV maturation and folding. Every single step mentioned utilizes host proteins and enzymes, which are the most important factors for DENV multiplications. Hence, targeting one of these can reduce DENV production and may lead to an effective antiviral drug.

The inflammatory response is activated in host cells during DENV infection to clear the pathogen from the host immune system. Whenever the host senses the presence of the DENV virus, activation of innate and inflammatory pathways occurs as a means of eliminating the disease. Alternation of host responses is a hallmark of dengue infection, whereby weak innate immunity and inflammatory response may lead to parasite growth and disease advancement. Again, excessive inflammation may be the reason behind the pathogenesis of severe dengue disease. In that case, a reduction of proinflammatory molecules can help to decrease dengue-induced vascular leakage. In summary, we require a better understanding of host innate and inflammatory pathways, as this information can help to identify appropriate targets. Targeting such inhibitors may result in antiviral drug development (focusing on blocking inflammation and endothelial barrier permeability) without interfering with the host immune mechanisms. Resolving the issues discussed in this work can yield more comprehensive knowledge about DENV and related host factors that can be utilized in novel therapeutic targets for the development of anti-DENV drugs.

## Figures and Tables

**Table 1:** List of DENV inhibitors isolated by targeting host process. LLC-MK2 = rhesus monkey kidney cell, BHK = baby hamster kidney, GAG = glycosaminoglycan, DC-SIGN = dendritic cell-specific intercellular adhesion molecule-3-grabbing non-integrin, MEK = MAPK/extracellular receptor kinase (ERK) kinase, NTRK1 = neurotrophic receptor tyrosine kinase 1, MAPKAPK = MAP kinase-activated protein kinase, RIG-I = retinoic-acid inducible gene I, IMP dehydrogenase = Inosine-5′-monophosphate dehydrogenase.

Host Process	Inhibitor(s)	Target	DENV Types	Cell Line(s) Tested	Refs.
Glycolytic pathway	2-deoxy-d-glucose (2DG)	Glycolysis	DENV-2	HFFs Cell	[5]
	Oxamate	Glycolysis	DENV-2	HFFs Cell	[5]
Lipid biosynthesis pathway	Cerulenin	Fatty acid biosynthesis	DENV-2	Huh-7.5 Cell	[156,157]
	C75	Fatty acid biosynthesis	DENV-4	C6/36 Cell	[156,157]
	Lovastatin (fluvastatin, lovastatin, mevastatin, and simvastatin)	Cholesterol biosynthesis	DENV-2	Huh-7 Cell	[158,159]
	U18666A	Cholesterol biosynthesis	DENV-2	C6/36 cell line	[160]
	Methyl b-cyclo dextrin	Cholesterol biosynthesis	DENV-1 to 4	Huh-7 Cell	[158]
	Nordihydroguaiaretic acid	Fatty acid biosynthesis	DENV-4	Huh-7 cell	[161]
	Orlistat	Fatty acid biosynthesis	DENV -4, -2	HepG2 and HEK293T/17 Cell	[161]
	PF-429242	Fatty acid biosynthesis	DENV-1–4	Huh-7.5.1 Cell	[162]
	Hymeglusin	Cholesterol biosynthesis	DENV-2	K562 cells	[68]
	Zaragozic acid	Cholesterol biosynthesis	DENV-2	K562 cells	[68]
Nucleotide biosynthesis pathways	Ribavirin	IMP dehydrogenase	DENV-2	LLC-MK2	[163]
	N-ally acridones	IMP dehydrogenase (Partial)	DENV-2	Vero cells	[163]
	Brequinar	Dihydroorotate dehydogenase	DENV-2	Vero cells	[164]
	Mycophenolic acid	IMP dehydrogenase	DENV-2	Huh-7, CRL-8024, and HepG2	[165]
	NITD 982	Dihydroorotate dehydogenase	DENV-2	Vero cells	[69]
	ETAR	IMP dehydrogenase	DENV-2	Vero cells	[166]
	IM18	IMP dehydrogenase	DENV-2	Vero cells	[166]
Glycosaminoglycans	PI88	Heparan sulfate	DENV-2	BHK and in mice	[80]
	Chondroitin sulfate	Heparan sulfate	DENV-1–4	BHK-21 and Vero cells	[78]
	Curdlan sulfate	Heparan sulfate	DENV-1–4	LLC-MK2 cells	[77]
	K5 polysaccharide from *Escherichia coli*	Heparan sulfate	DENV-2	HMEC-1 and HMVEC-d cells	[83]
	Heparin	Heparan sulfate	DENV-2	Vero, BHK, Hepatocytes	[79]
	Fucoidans	Heparan sulfate	DENV-2	BHK	[82]
	GAG	Heparan sulfate	DENV-2	Vero	[79]
	Sulfated galactomannan	Heparan sulfate	DENV-1	C6/36	[167]
	DL-galactan	Heparan sulfate	DENV-2, -3	Vero, Hep-G2	[141]
	Carrageenan	Heparan sulfate	DENV-2, -3	Vero, Hep-G2	[138]
	α-d-glucan	Heparan sulfate	DENV-2	BHK	[84]
	Dextran sulfate 8000	Heparan sulfate	DENV-2	Hepatocytes, Vero	[168]
	Zosteric acid, CF-238	Heparan sulfate	DENV-1–4	LLC-MK2	[77]
DC-SIGN	PRM-S	Carbohydrate binding agent	DENV-2	Raji/DC-SIGN and MDDC	[169]
	QL-XII-47 (QL47)	DC-SIGN(BTK)	DENV-2	Huh-7 Cell	[170]
	Plant lectins from *Hippeastrum* hybrid, *Galanthus nivalis*, *Urtica dioica*	DC-SIGN	DENV-1–4	MDDC, Huh-7, U87/DC-SIGN	[169]
	Glycomimetic DC-SIGN ligand	DC-SIGN	DENV-2	DC-SIGN/Raji cells	[171]
	DS (MW > 500,000 Da)	DC-SIGN	DENV-1–4	C6/36	[169]
Host protease	45	Furin	DENV-2	Huh-7 cells	[172]
	46	Furin	DENV-2	Huh-7 cells	[172]
	Peptidomimettic furin inhibitor, Luteolin	Furin	DENV-1–4	Huh-7 cells	[105]
Host kinase	Dasatinib	c-Src/Fyn	DENV-1–4	Vero, Huh-7	[106]
	SaracatinibAZD0530	c-Src/Fyn	DENV-1–4	Vero, Huh-7	[106]
	GNF-2	Abl Kinases E Protein	DENV-2	BHK-21	[108]
	Imatinib	Abl Kinases	DENV-2	BHK-21	[108]
Mitogen activated protein kinase	PD98059, U0126, FR180204	MEK	DENV-2	RAW264.7	[173]
	SB203580	p38 pathway	DENV-2	C6/36	[174]
	CGP57380	ERK and p38 pathways	DENV-2	BHK-21	[175]
	Imidazo[1,2-b] pyridazine	AAK1	DENV-2	Huh-7	[176]
	Isothiazolo[5,4-b] pyridines	GAK	DENV-2	Huh-7	[177]
	Sunitinib and erlotinib	AAK1 and GAK	DENV-2	Huh7	[178]
	AR-12	PI3K/JAKT pathway	DENV-1–4	Huh 7	[179]
	U0126	Erk inhibitor	DENV-2, -3	BHK-21	[180]
	SFV785	NTRK1 and MAPKAPK5	DENV-2	BHK-21	[108]
Host Glucosidase	CM-9-78 (DNJ derivative)	α-glucosidase	DENV-1 to 4	BHK-21	[181]
	UV-4 (DNJ derivative)	α-glucosidase	DENV-2	BHK-21	[117]
	DNJ	α-glucosidase	DENV-1	BHK-21	[117]
	Celgosivir	α-glucosidase	DENV-1–4	BHK	[116]
	Kotalanol	α-glucosidase	DENV-1–4	BHK	[86]
	Castanospermine	α-glucosidase	DENV-1–4	BHK, Huh-7	[110,112]
	OSL-9511	α-glucosidase	DENV-2	BHK	[182]
	NN-DNJ	α-glucosidase	DENV-2	BHK	[114]
	Compound 36	α-glucosidase	DENV-2	BHK-21	[183]
	Compound 36	α-glucosidase	DENV-2	BHK-21	[183]
	Compound 36	α-glucosidase	DENV-2	BHK-21	[183]
	N-alkyl side chains 69(CST)	α-glucosidase	DENV-2	BHK	[183]
	N-alkyl side chains 70(DNJ)	α-glucosidase	DENV-2	BHK	[183]
	N-alkyl side chains 71	α-glucosidase	DENV-2	BHK	[182]
	N-alkyl side chains 72	α-glucosidase	DENV-2	BHK	[182]
	N-alkyl side chains 73	α-glucosidase	DENV-2	BHK	[182]
	N-alkyl side chains 74	α-glucosidase	DENV-2	BHK	[184]
	N-alkyl side chains 75	α-glucosidase	DENV-2	BHK	[184]
	N-alkyl side chains 76	α-glucosidase	DENV-2	BHK	[184]
	N-alkyl side chains 77	α-glucosidase	DENV-2	BHK	[185]
	N-alkyl side chains 78	α-glucosidase	DENV-2	BHK	[117]
	N-alkyl side chains 79	α-glucosidase	DENV-2	BHK	[186]
	SP173	α-glucosidase	DENV-2	BHK-21	[112]
	SP169	α-glucosidase	DENV-2	BHK-21	[112]
	6-*O*-butanoyl castanospermine	α-glucosidase	DENV-2	BHK-21	[110]
Host Immunity, and Inflammatory pathways	Human heme oxygenase I	Innate antiviral response	DENV-1–4	Huh-7	[187]
	Schisandrin A	STAT1/2-mediated responses	DENV-1–4	Huh-7	[188]
	Celastrol	JAK–STAT signaling	DENV-1–4	Huh-7	[189]
	Agonists of IRF3-terminal pathways	TRIF Pathway	DENV-2	Vero cells	[190]
	Salidroside	RIG-I	DENV-2	THP-1 cell line	[191]
	Asunaprevir	MAVS pathways	DENV-2	Huh 7.5.1, Hep-G2 cells,	[192]
	Sequence-specific RIG-I agonist	IRIG-I-mediated	DENV-2	Lung epithelial A549 cells	[193]
	Helicase with zinc linger 2	Innate antiviral response	DENV-2	Vero cells	[194]
	Purinergic receptor P2X7	Inflammatory process	DENV-2	Human monocyte Cell	[195]
	Extract from *Uncaria tomenrosa*, *N. brasiliensis Choisy*, *Uncaria guianensis*	Cytokine/chemokine	DENV-2	Huh-7	[196,197]
	Extract from *Cissampelos pareira Linn*	Innate antiviral response	DENV-1–4	C6/36, LLC-MK2, Vero, Hep-G2	[198]
	Ivermectin	α/β-mediated transport	DENV-1–4	HeLa	[198]
	BST2/tetherin	IFN *induced*	DENV-2	Huh7	[199]
